# MiR-873-5p: A Potential Molecular Marker for Cancer Diagnosis and Prognosis

**DOI:** 10.3389/fonc.2021.743701

**Published:** 2021-10-05

**Authors:** Yuhao Zou, Chenming Zhong, Zekai Hu, Shiwei Duan

**Affiliations:** ^1^Institute of Translational Medicine, Zhejiang University City College, Hangzhou, China; ^2^Medical Genetics Center, Ningbo University School of Medicine, Ningbo, China; ^3^Department of Clinical Medicine, Zhejiang University City College School of Medicine, Hangzhou, China

**Keywords:** miR-873-5p, cancer, cell, prognosis, signaling pathway

## Abstract

miR-873 is a microRNA located on chromosome 9p21.1. miR-873-5p and miR-873-3p are the two main members of the miR-873 family. Most studies focus on miR-873-5p, and there are a few studies on miR-873-3p. The expression level of miR-873-5p was down-regulated in 14 cancers and up-regulated in 4 cancers. miR-873-5p has many targeted genes, which have unique molecular functions such as catalytic activity, transcription regulation, and binding. miR-873-5p affects cancer development through the PIK3/AKT/mTOR, Wnt/β-Catenin, NF-κβ, and MEK/ERK signaling pathways. In addition, the target genes of miR-873-5p are closely related to the proliferation, apoptosis, migration, invasion, cell cycle, cell stemness, and glycolysis of cancer cells. The target genes of miR-873-5p are also related to the efficacy of several anti-cancer drugs. Currently, in cancer, the expression of miR-873-5p is regulated by a variety of epigenetic factors. This review summarizes the role and mechanism of miR-873-5p in human tumors shows the potential value of miR-873-5p as a molecular marker for cancer diagnosis and prognosis.

## Introduction

**Figure 1:**
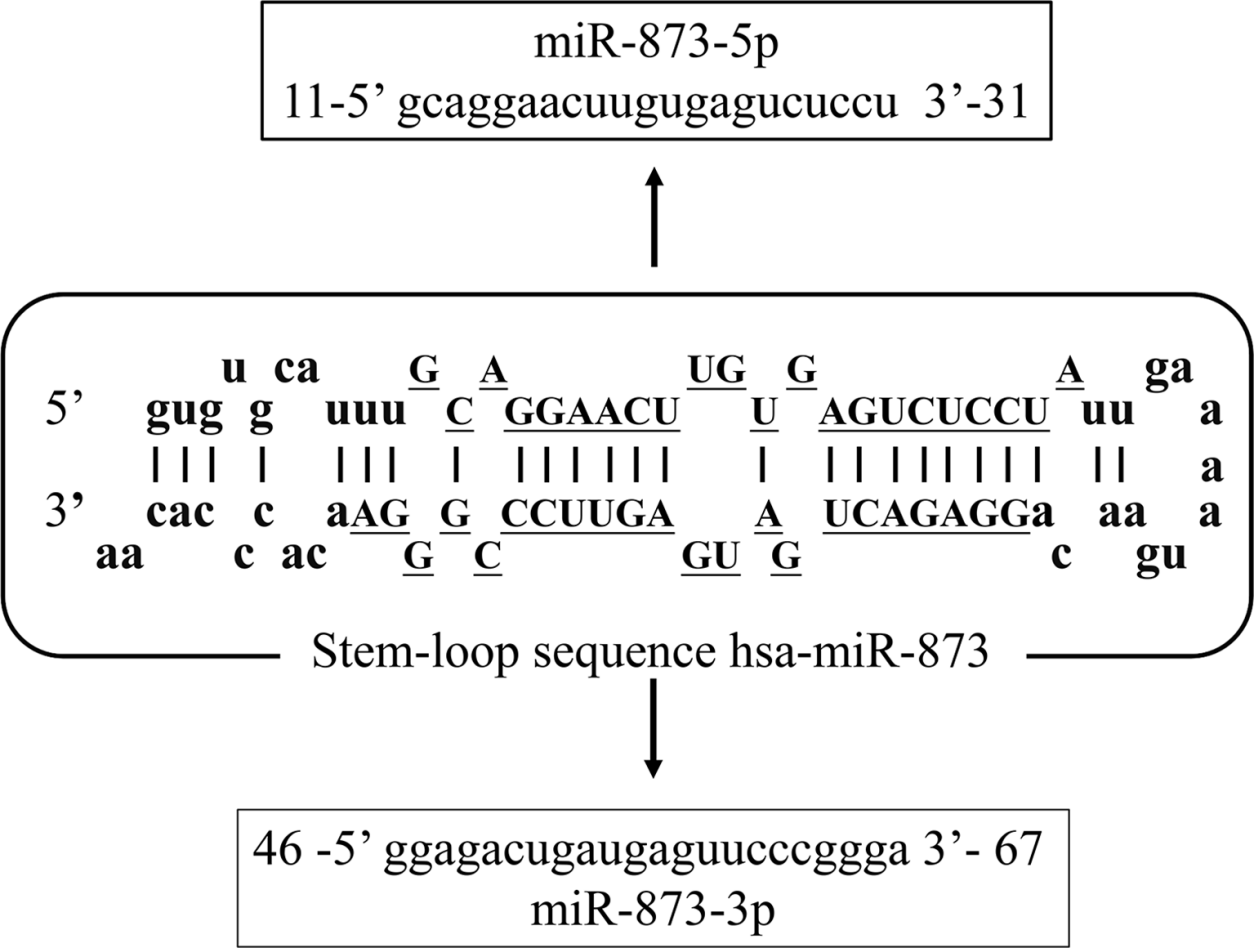
The sequence structure of the miR-873 family. Hsa-mir-873 is located on chromosome 9 (chr9:28888878-28888954). It has two mature sequences, hsa-miR-873-5p (MIMAT0004953, miR-873-5p) and hsa-miR-873-3p (MIMAT0022717, miR-873-3p).

With the increasing incidence and mortality of cancer worldwide in recent decades, it has become the second leading cause of human death (1). MicroRNA (miRNA) is a set of non-coding RNA (2) less than 25 nucleotides in length. miRNAs can bind to the 3’-untranslated region (3’-UTR) of target mRNA molecules and regulate the expression of target genes, thus playing an important role in cancer (3). The miR-873 family is located on chromosome 9 (chr9:28888878-28888954). Its family includes two main members of the human genome, including hsa-miR-873-5p (miR-873-5p) and hsa-miR-873-3p (miR-873-3p). Their mature sequences are 21 and 22 nucleotides in length, respectively, and are highly conserved (**Figure 1**). At present, most researches focus on miR-873-5p.

Studies have found that the expression of miR-873-5p is dysregulated in a variety of cancers and plays different roles in different cancers. On the one hand, miR-873-5p is upregulated and carcinogenic in non-small cell lung cancer (NSCLC) (4), and hepatocellular carcinoma (HCC) (5); on the other hand, miR-873-5p is involved in colorectal cancer (CRC) (6) and gastric cancer (GC) (7) are down-regulated and exert a tumor suppressor effect. miR-873-5p can affect cell proliferation (5), apoptosis (6), migration (8), invasion (9), cell stemness (10), and other biological processes by regulating the expression of its target genes. In addition, miR-873-5p also has important clinical significance in drug sensitivity and prognosis of cancer patients (4, 6). miR-873-5p can also be regulated by a variety of epigenetic factors. Among them, the interaction between non-coding RNA (lncRNA or circRNA) and miR-873-5p is mainly researched. This review focuses on studying the biological role of miR-873-5p in tumors, exploring the molecular functional network of its targeted genes, and predicting the potential role of miR-873-5p in the diagnosis and prognosis of human cancer.

## The Biological Function of miR-873-5p Target Genes

**Figure 2:**
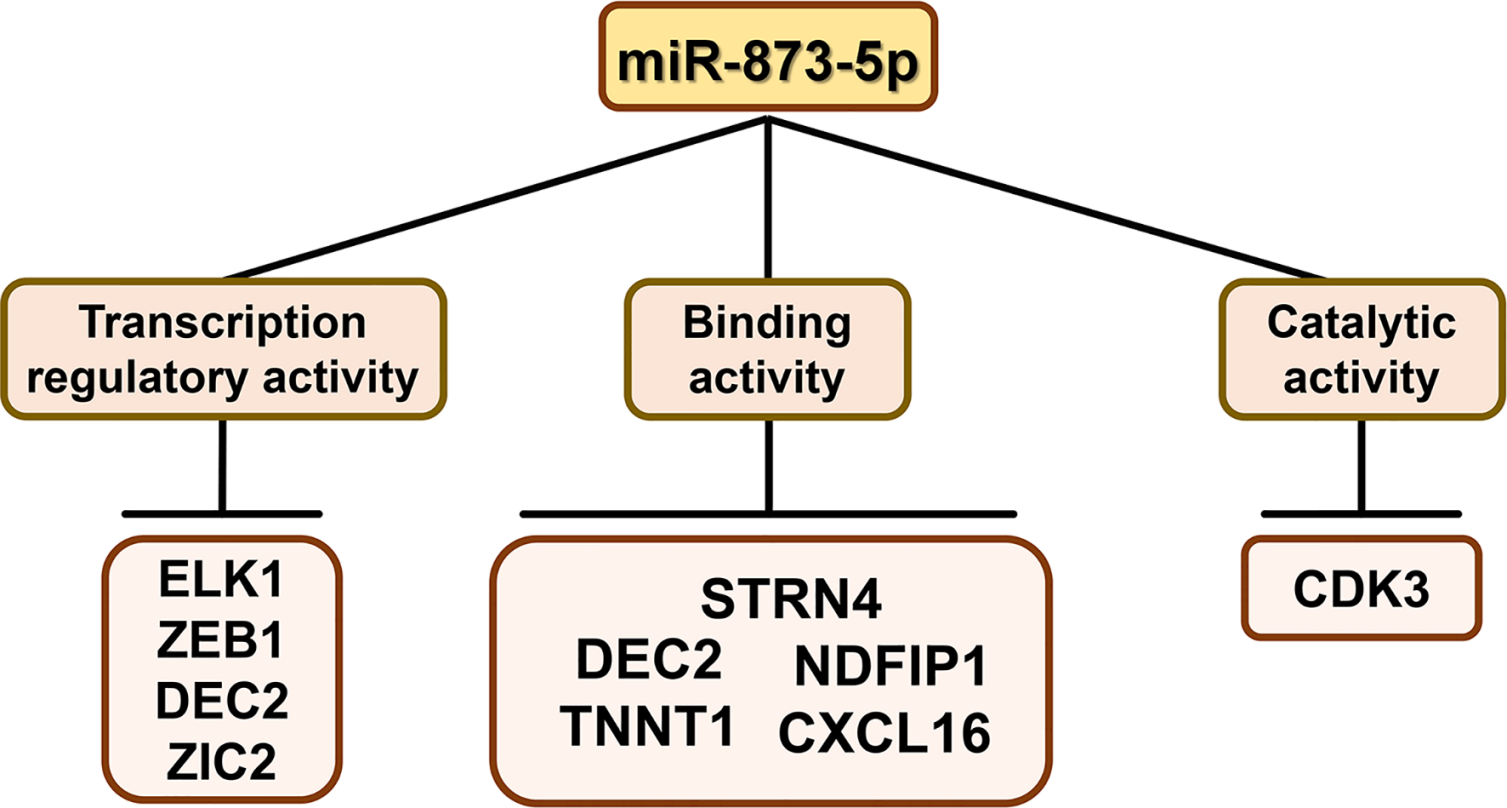
The molecular functions of miR-873-5p target genesThe target genes of miR-873-5p have the molecular functions of binding, catalytic, and transcription regulator activity.

miR-873-5p can directly bind to the 3’-UTR of target gene mRNA and regulate gene expression after transcription. The target gene of miR-873-5p has unique molecular functions, including catalytic activity, transcription regulation, binding, etc. (**Figure 2**).

Among the miR-873-5p target genes, CDK3 is a catalytically active gene. CDK3 is a cyclin-dependent kinase, which can phosphorylate the estrogen receptor (ER) and enhance ER activity, thereby promoting the occurrence and development of breast cancer (BC) (11).

Among the miR-873-5p target genes, genes with transcriptional regulatory activity are ELK1, DEC2, ZEB1, and ZIC2. ELK1 is a key transcriptional regulator that mediates the MEK-ERK signal transduction, and it can activate early oncogene expression (12, 13). DEC2 is the basic helix-loop-helix transcription factor of the clock gene. It plays an important role in the circadian rhythm, cell proliferation, and apoptosis, and thus participates in tumor progression (14). ZEB1 is a member of the zinc finger E-box binding protein (ZEB) transcription factor family (15). ZEB1 can bind to the promoter of the liver cancer-derived growth factor (HDGF) and increase the level of HDGF transcription, leading to the pathogenesis of endometrial cancer (EC) (16). ZIC (Cerebellar Zinc Finger Protein) protein has five highly conserved Cys2His2 motifs, which can bind to DNA and thus function as a transcription factor (17). As a member of the ZIC family, ZIC2 can promote tumor growth and metastasis of hepatocellular carcinoma through transcriptional regulation of p21-activated kinase 4 (18). In addition, ZIC2 can bind to the DNA-binding high mobility base box of TCF4, thereby inhibiting the transcriptional activity of β-catenin (19).

The miR-873-5p target genes with binding activity include DEC2, NDFIP1, STRN4, TNNT1, and CXCL16. DEC2 can inhibit its downstream molecules by binding to the E-box (20). NDFIP1 is a membrane protein with small endosomes containing PY motifs, which can transport E3 ligase and its substrate to endosomes (21). STRN4 is a member of the striatin family. It can combine with MINK1 of the germinal center kinase family to form a large complex, which is essential for the process of cytokinesis (22, 23). Troponin T1 (TNNT1) is a subunit of troponin T, which can bind to tropomyosin and anchor the troponin complex at a specific location on striated muscle filaments (24). CXCL16-CXCR6 are chemokines and chemokine receptors, respectively, which can bind to each other (25). The mutual binding of CXCL16 and CXCR6 involves a variety of biological activities, including cell adhesion (26) and anti-tumor immunity (27).

## MiR-873-5p Dysregulation in Various Cancers

As shown in **Table 1**, miR-873-5p is abnormally expressed in 18 types of cancers. Among them, miR-873-5p is up-regulated in 4 types of cancers, including NSCLC (4), lung adenocarcinoma (LUAD) (28, 29), lung cancer (LCA) (5, 8, 30), and Merkel cell carcinoma (MCC) (24627810). miR-873-5p is down-regulated in 14 types of cancers, including nasopharyngeal carcinoma (NPC) (32), lung cancer (LCA) (33), cervical cancer (CC) (34, 35), EC (36), BC (10, 11, 37, 38), pancreatic ductal adenocarcinoma (PDAC) (39), glioblastoma (GM) (9, 40–42), osteosarcoma (OS) (43), papillary thyroid carcinoma(PTC) (44), CRC (6, 45–49), esophageal cancer(ESCA) (50),GC (7, 51, 52), tongue squamous cell carcinoma (TSCC) (53), and pancreatic cancer(PC) (54).

**Table 1:** miR-873-5p dysregulation and its target genes in cancer.

Cancer type	Clinical Samples	Cell lines (Cancer cells and Normal cells)	*In vitro*	*In vivo*	Expression	Target gene	Reference
NSCLC		PC9 and BEAS-2B, HEK293T	Proliferation↑		Upregulation	GLI1	(4)
LUAD	30 LUAD tissues and 30 matched non-tumor tissues	H23, H1299, A549, SPC-A1	Proliferation↑; migration and invasion↑		Upregulation	SRCIN1	(28)
	481 LUAD tissues and 47 normal tissues				Upregulation	—	(29)
HCC	86 HCC tissues and 86 matched non-tumor tissues	SMMC-7721, HepG2, Hep3B, SK-HEP-1, MHCC97H and L02, 7701, 7702	Proliferation↑; glycolytic metabolism↑		Upregulation	NDFIP1	(5)
	25 HCC tissues and 25 adjacent non-tumor tissues	HuH6, THLE-2 and ATCC, Manassas, VA, USA	Proliferation↑; migration and invasion↑		Upregulation	TRIM25	(8)
	70 HCC tissues and 70 adjacent non-tumor tissues	Hep3B, HepG2, SMMC-7721, Huh-7 and L02	Proliferation↑; migration and invasion↑		Upregulation	TSLC1	(30)
MCC	3 MCC tissues, 1 SCC tissue, 1 BCC tissue,1 normal skin				Upregulation	—	(31)
NPC	134 NPC tissues and 40 non-NPC tissues	5-8 F, 6-10B, HNE-3, C666-1 and NP69SV40T	Cell stemness↓		Downregulation	ZIC2	(32)
LCA	31 NSCLC tissues and 31 matched normal tissues		Cell stemness↓		Downregulation	CDK3	(33)
CC	306 CC tissues and 3 normal tissues	Caski, HeLa, C33a, SiHa	Proliferation↓		Downregulation	ULBP2	(34)
	20 CC tissues and 20 matched normal tissues	C33A, HeLa, SiHa and Ect1/E6E7	Proliferation↓; migration and invasion↓; EMT↓		Downregulation	GLI1	(35)
EC	47 EC tissues and 47 adjacent non-tumor tissues	AN3CA, HEC-59, HEC-1B, KLE and HUM-CELL-0111	Proliferation↓		Downregulation	HDGF	(36)
BC	4 BC tissues and 4 adjacent mammary gland epithelial tissues		Cell stemness↓	Cell stemness↓	Downregulation	PD-L1	(10)
	43 BC tissues and 10 adjacent non-tumor tissues	MCF-7, ZR75-1, T47D, SKBR3, MDA-MB-231 and HEK293T	Proliferation↓	Tumor growth↓	Downregulation	CDK3	(11)
		MDA-MB-231, BT549 and 293	——		Downregulation	ZEB1	(37)
	30 TNBC tissues and 30 adjacent normal tissues	MDA-MB-453, BT-549, MDA-MB-231, HCC1937 and HBL-100	Proliferation↓; migration and invasion↓; EMT↓		Downregulation	DCST1-AS1	(38)
PDAC/TNBC		MDA-MB-436, MDA-MB-231, MDA-MB-453, BT-20, HCC1937, SKBR3, T47D, HEK293 and HPDE	Proliferation↓; migration and invasion↓	Proliferation↓; tumor growth↓	Downregulation	KRAS	(39)
GM	6 GM tissues and 3 non-tumor brain tissue		——		Downregulation	—	(40)
	12 high-grade GM tissues and 7 normal brain tissues	U87, U251	——		Downregulation	Bcl-2	(9)
	50 GM tissues and 50 normal tissues		——		Downregulation		(41)
	6 GBM tissues and 6 adjacent normal tissues	A172, T98G, U87, U373, U251, U138	Proliferation↓; migration and invasion↓; apoptosis↑	apoptosis↑	Downregulation	IGF2BP1	(42)
OS	49 OS tissues and 49 adjacent normal bone tissues	MG-63, SAOS-2, HOS, U2OS and hFOB1.19	Proliferation↓; migration and invasion↓	Tumor growth↓	Downregulation	HOXA9	(43)
PTC	30 PTC tissues and 30 adjacent normal tissues	KTC-1, TPC-1, BCPAP, K1, BHP10-3 and Nthy-ori3-1	Proliferation↓; migration and invasion↓		Downregulation	CXCL16	(44)
CRC	50 CRC tissues and 50 adjacent normal tissues	HCT116, H29, SW620, LOVO, SW480 and NCM460	Proliferation↓; migration and invasion↓; EMT↓; apoptosis↑; cell cycle↑		Downregulation	JMJD8	(6)
	10 CRC tissues and 10 adjacent non-tumor tissues	SW620, SW480, DLD1, HCT116, LoVo, HT-29 and NCM460	Proliferation↓		Downregulation	TRAF5/TAB1	(45)
		DLD-1, HCT-116, SW-480, HT-29, SW-620 and HIEC	Migration and invasion↓; EMT↓		Downregulation	ZEB1	(46)
	55 CRC tissues and 55 adjacent normal tissues	SW620, HCT116, HCT8, SW480, LS174T, HT29, RKO	Proliferation↓; migration and invasion↓; EMT↓	Cell growth↓; liver metastasis↓	Downregulation	ELK1/STRN4	(47)
	45 CRC tissues and 45 adjacent normal tissues	HT29, SW480, HCT116 and CRL1790	Proliferation↓; migration and invasion↓		Downregulation	TNNT1	(48)
	96 CC tissues and 96 adjacent normal tissues	HCT116, SW620, RKO, HCT8, HT29 and NCM460	Proliferation↓; migration and invasion↓; EMT↓	Proliferation↓; metastasis↓	Downregulation	TUSC3	(49)
ESCA	36 EC tissues and 36 adjacent normal tissues	EC-109, EC-1, TE-1, TE-10, KYSE-150 and HEEC	Proliferation↓; migration and invasion↓; EMT↓		Downregulation	DEC2	(50)
GC	80 GC tissues and 80 adjacent non-tumor tissues	SGC-7901	Proliferation↓; apoptosis↑; cell cycle↑		Downregulation	GLI1	(51)
	15 GC tissues and 15 adjacent non-tumor tissues and 15 normal tissues				Downregulation	—	(7)
	80 GC tissues and 80 adjacent normal tissues	BGC823, SGC7901, MKN45, MGC803 and GES-1	Proliferation↓; migration and invasion↓; EMT↓; cell cycle↑	Proliferation↓; metastasis↓	Downregulation	STRA6	(52)
TSCC	35 TSCC tissues and 35 adjacent normal tissues	SCC9, SCC15, SCC25, UM1, CAL-27 and HOEC	Apoptosis↑		Downregulation	SEC11A	(53)
PC	30 PC tissues and 45 normal tissues	PANC-1, SW1990, MIA PaCa-2 and hTERT-HPNE	Cell stemness↓		Downregulation	PLEK2	(54)

NSCLC, non-small cell lung cancer; LUAD, lung adenocarcinoma; HCC, hepatocellular carcinoma; MCC, Merkel cell carcinoma; LCA, lung cancer; CC, cervical cancer; EC, endometrial cancer; BC, breast cancer; GM, glioblastomas; TNBC, triple-negative breast cancer; PDAC, pancreatic ductal adenocarcinoma; PTC, papillary thyroid cancer; CRC, carcinoma of colon and rectum; GC, gastric cancer; ESCA, esophageal cancer; OS, osteosarcoma; NPC, nasopharyngeal carcinoma; PC, pancreatic cancer.

↑: promotion; ↓: inhibition.

Highly expressed miR-873-5p can inhibit cell proliferation, induce cell apoptosis, inhibit EMT, metastasis, and invasion process, thereby promoting the occurrence and development of cancer. Among the four types of cancers (NSCLC, LUAD, HCC, and MCC), miR-873-5p can promote their progression, indicating that miR-873-5p has tumor suppressor and cancer-promoting effects.

## The Biological Role of miR-873-5p in Human Cancer

### MiR-873-5p and Different Signaling Pathways

**Figure 3:**
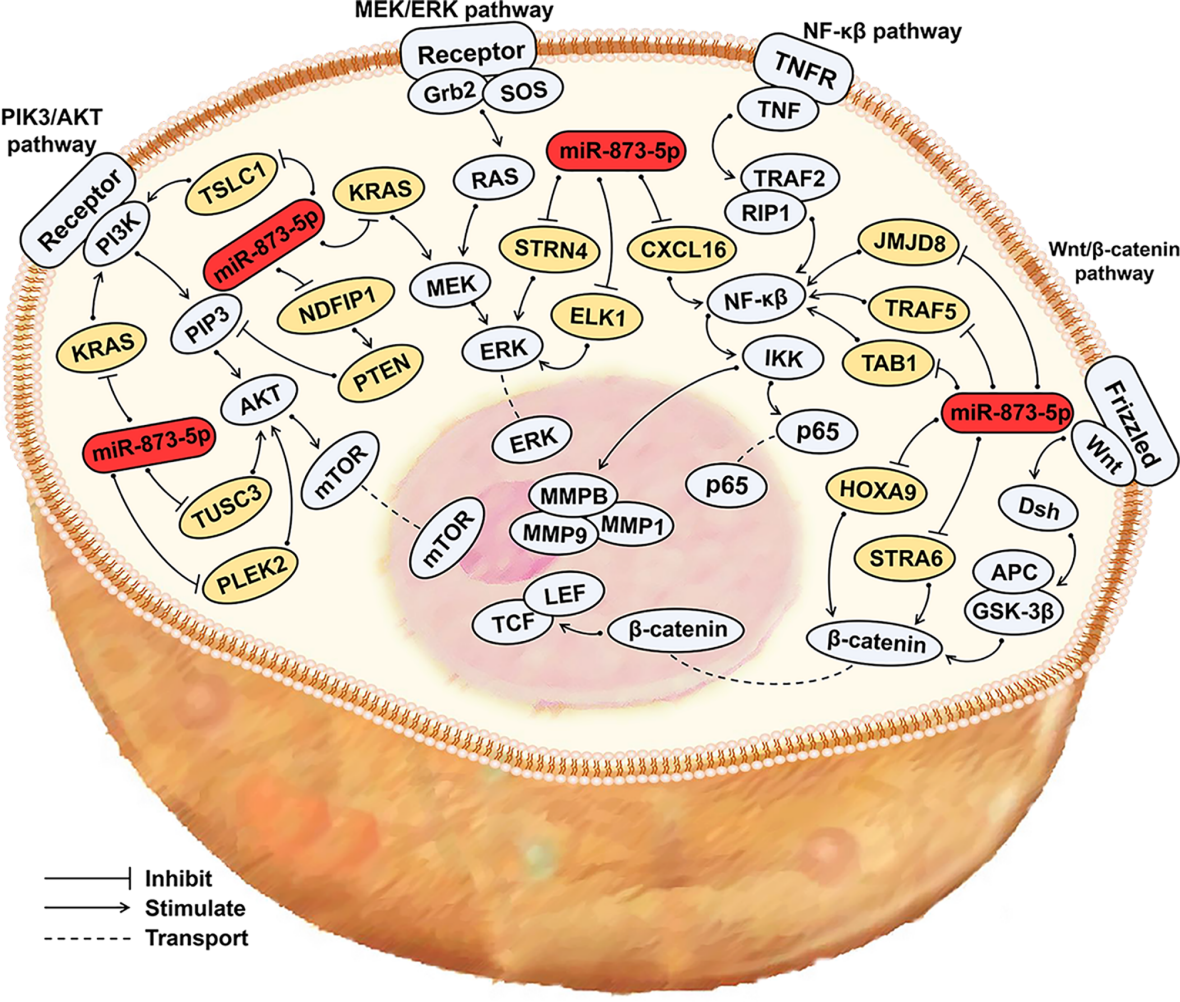
miR-873-5p related signaling pathways in cancer. miR-873-5p can influence cancer development by participating in the PIK3/AKT/mTOR, Wnt/β-Catenin, NF-κβ, MEK/ERK signaling pathways. Blue, signaling pathway; Orange, target gene; Red, miR-873-5p.

miR-873-5p can affect the occurrence and development of cancer by participating in the PIK3/AKT/mTOR, Wnt/β-Catenin, NF-κβ, MEK/ERK, and other signaling pathways (**Figure 3**).

The PIK3/AKT signaling pathway is often overactivated in malignant tumors. The PIK3/AKT signaling pathway can participate in cell cycle regulation, promote cell proliferation and metastasis, and inhibit cell apoptosis (55). In HCC, miR-873-5p promotes the development of HCC through the NDFIP1/AKT/mTOR axis (5). miR-873-5p can directly activate PIK3/AKT to promote HCC progression (30). miR-873-5p can down-regulate TUSC3 expression, inhibit the AKT signaling pathway, and thus hinder CRC development (49). In PC, miR-873-5p targets PLEK2 and inhibits the AKT signaling pathway, thereby inhibiting the development of cancer (54).

The Wnt/β-Catenin signaling pathway is important for tumor development, and the dysregulation of the Wnt/β-Catenin signaling pathway may lead to cell proliferation and malignancy (56). miR-873-5p inhibits the expression of HOXA9 and STRA6, and blocks the Wnt/β-Catenin signaling pathway, thereby inhibiting the development of OS and GC (43, 52).

The NF-κβ signaling pathway can inhibit cell apoptosis, and it is closely related to tumor occurrence, growth, and metastasis (57). By inhibiting the expression of JMJD8, TNF receptor-related factor 5 (TRAF5) and TGF-β activated kinase 1 (MAP3K7) binding protein 1 (TAB1), miR-873-5p can inhibit the NF-κβ signaling pathway, thereby hindering the progression of CRC (6, 45). In PTC, miR-873-5p can down-regulate the expression of CXCL16, and can also inhibit the development of PTC through down-regulating the NF-κβ signaling pathway (44).

The MEK/ERK signaling pathway can promote cell proliferation and migration and is involved in the occurrence and development of a variety of cancers (12). In CRC, miR-873-5p targets ELK1 and STRN4, and exerts a tumor suppressor effect through the ERK signaling pathway (47). By down-regulating KRAS expression, miR-873-5p can also inhibit the ERK signaling pathway to suppress the development of PDAC and TNBC (39). In addition, miR-873-5p can also deactivate the PI3K/AKT and ERK signaling pathways to inhibit the development of BC (58).

### MiR-873-5p and Cell Cycle

**Figure 4:**
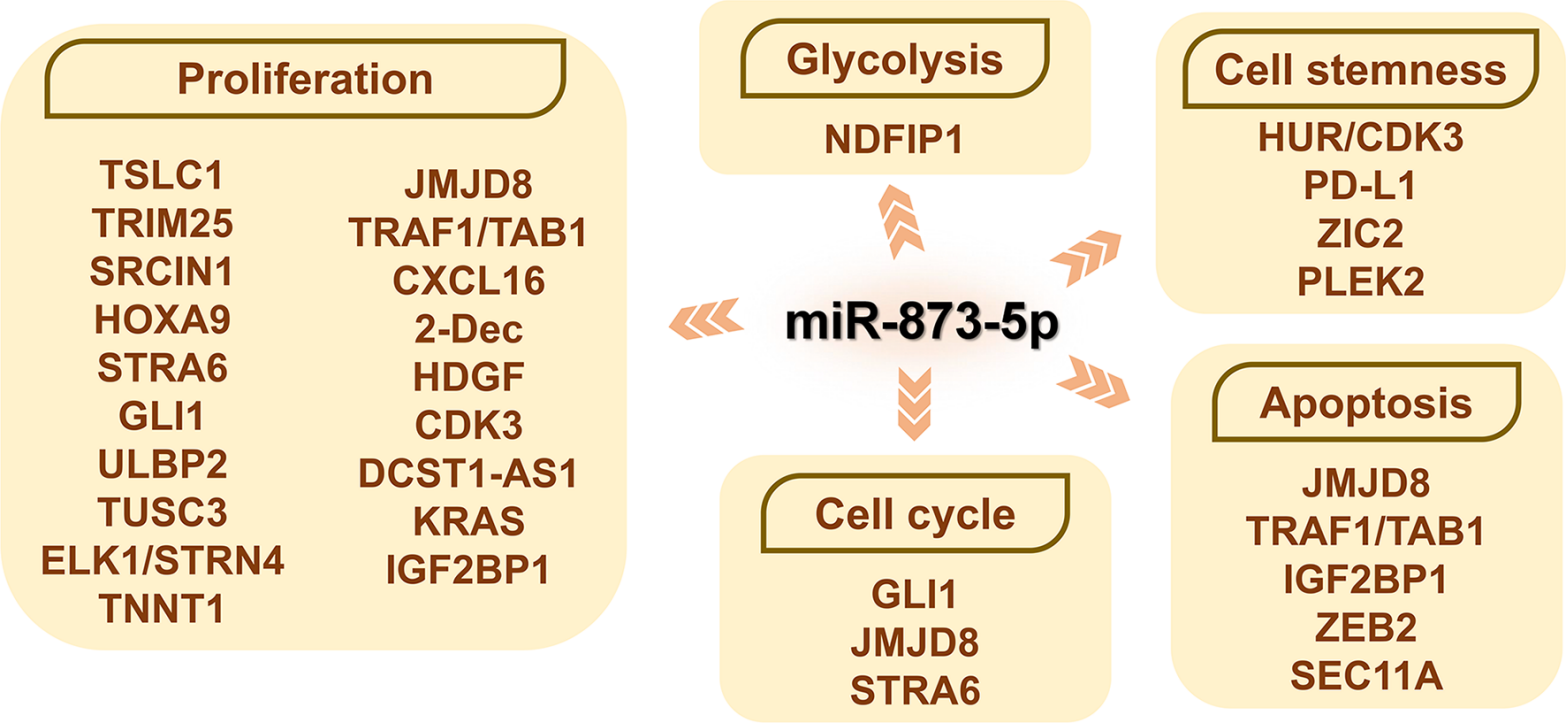
The role of miR-873-5p and its target genes on the cell biology of cancer cells. By promoting or inhibiting cell proliferation and apoptosis, miR-873-5p has both oncogenic or pro-cancer effects in different cancers. miR-873-5p inhibits the aerobic glycolysis of cancer cells by targeting NDFIP1. In addition, miR-873-5p can reduce the stemness of cancer cells by targeting PD-L1 and HUR/CDK3. By targeting GLI1, JMJD8, IGF2BP1, and STRA6, miR-873-5p can inhibit the progression of the cancer cell cycle.

The regulation of the cell cycle is of great significance to the proliferation and apoptosis of cancer cells (**Figure 4**). Increased expression of miR-873-5p can inhibit the expression of GLI1 and cyclin B, thereby inducing GC cells to arrest the G2/M cell cycle (51). After miR-873-5p targets to inhibit JMJD8, it blocks CRC HCT116 and SW480 cells in the G1-S cell cycle (6). miRNA-873-5p can accelerate the S phase process of HCC cells, thereby promoting cancer cell proliferation (30). Other studies have shown that miR-873-5p can down-regulate STRA6, thereby inducing GC cells to arrest in the G0/G1 cell cycle and increasing cell mortality (30). After miR-873-5p targeted IGF2BP1, GM cells showed significant G0/G1 block and S phase reduction (42).

### MiR-873-5p and Cell Proliferation and Apoptosis

The targeted genes of miR-873-5p are closely related to the process of tumor cell proliferation and apoptosis (**Figure 4**).

STRN4 directly acts on protein kinases such as MINK1, TNIK, and MAP4K4. The knockdown of STRN4 inhibits the proliferation of PDAC and CRC cancer cells (59). miR-873-5p can target ELK1 and STRN4 and inhibit the proliferation of CRC LoVo and HCT116 cells through the regulation of the ERK-CyclinD1 signaling pathway (47).

Human cytomegalovirus glycoprotein UL16 binding protein 2 (ULBP2) is an important activation receptor on the surface of natural killer cells. In normal tissues, low levels of ULBP2 can lead to the activation of immune cells (60, 61). In CC C33a cells, miR-873-5p activates immune cells by inhibiting ULBP2 expression, thereby attenuating cell proliferation (34).

Jumonji domain-containing protein 8 (JMJD8) contains a JmjC domain (62) at 74-269 amino acid residues. miR-873-5p can inhibit the NF-κβ signaling pathway by down-regulating the expression of JMJD8 in CRC cells, thereby inhibiting cell proliferation, blocking the G1-S transition, and enhancing the apoptosis of CRC HCT116 and SW480 cells (6). miR-873-5p directly targets the 3’-UTR of TUSC3 to down-regulate its expression and inhibit AKT signaling pathway and CRC cell proliferation (49). TNNT1 expression is closely related to the clinical stage of tumor tissues and can promote the proliferation of cancer cells through metastatic G1/S transition (63). miR-873-5p down-regulates TNNT1 and may inhibit the proliferation of CRC cells (48). Besides, TRAF5 and TAB1 are both key components of the NF-κβ signaling pathway (45). miR-873-5p directly targets TRAF5 and TAB1 to inhibit the NF-κβ signaling pathway, thereby inhibiting the cell proliferation of CRC (45).

KRAS can enhance the AKT and ERK signaling pathways that are related to cell proliferation (64). miR-873-5p inhibits the cell proliferation of PDAC and TNBC tissues (38) by targeting KRAS, thereby inhibiting the ERK and PI3K/AKT signaling pathways (39). miR-873-5p can induce apoptosis of PDAC and TNBC by regulating the Caspase-dependent apoptotic pathway (39). DCST1-AS1 is an oncogenic lncRNA (38). DCST1-AS1 can sponge miR-873-5p and thus reduce the inhibition of miR-873-5p on the expression of IGF2BP1, thereby up-regulating the expression of MYC and promoting the proliferation of TNBC cells (38).

Tripartite motif-containing protein 25 (TRIM25) is a member of TRIM protein, which can target the degradation of MTA-1 (65). MTA-1 is a member of the metastasis-related gene (MTA) family and plays an important role in the proliferation of cancer cells (66). miR-873-5p can inhibit TRIM25 expression, which can promote the proliferation of HCC cells (8). TSLC1 is a new type of tumor suppressor gene, which is related to proliferation, apoptosis, cell cycle, and tumorigenicity of cancer cell (67). The inhibition of TSLC1 by miRNA-873-5p can lead to hyperphosphorylation of PI3K/AKT/mTOR and other signaling pathways to promote HCC cell proliferation (30).

Src is a tyrosine kinase that is frequently up-regulated in cancer and is very important for cancer cell proliferation (68, 69). Src Kinase Signaling Inhibitor 1 (SRCIN1) is a tumor suppressor gene that suppresses cancer by inactivating Src in cancer (70). miR-873-5p activates the Src signaling pathway by down-regulating of SRCIN1 expression and promotes the proliferation of LUAD cells (28).

Insulin-like growth factor 2 mRNA binding protein 1 (IGF2BP1) is a carcinoembryonic protein that is expressed in various cancers including leukemia (71). IGF2BP1 can stabilize and enhance the expression of c-MYC and MKI67, which are both effective regulators of cell proliferation and apoptosis (72). Overexpression of miR-873-5p in GM cells can significantly down-regulate the expression of IGF2BP1, MKI67, and c-MYC, and lead to cell proliferation inhibition and apoptosis (42). ZEB2 is a transcription factor containing zinc fingers, which is essential in early embryonic development (73). ZEB2 can increase the expression of cyclin A1, cyclin D1, and Bcl-2 in GM cells, thereby promoting the growth of GM cells (74). miR-873-5p down-regulates ZEB2 expression, which can promote GM cell apoptosis (74).

HOXA9 is a member of the mammalian HOX family (75), which is abnormally activated in a variety of cancers such as CRC (76) and GC (77). miR-873-5p directly targets HOXA9 and reduces the expression levels of β-catenin and cyclin D1 through the inactivation of the Wnt/β-catenin signaling pathway, thereby inhibiting OS cell proliferation (43).

Hedgehog (Hh) signaling pathway can participate in the cancer process through mechanisms such as promotion of tumor invasion and metastasis (78, 79). GLI1 is a transcription factor of the Hh signaling pathway and downstream target genes and is usually used as a marker to activate the Hh signaling pathway (80). Studies have found that increased expression of miR-873-5p can inhibit the expression of GLI1 and inhibit the cell proliferation of NSCLC (4), GC (51), and CC (35) through the Hh signaling pathway. STRA6, as a transmembrane protein of RA, is overexpressed in many cancer types (81). Overexpression of STRA6 can upregulate Wnt pathway-related genes, such as β-catenin, MMP-7, and c-myc. miR-873-5p down-regulates the expression of STRA6 in GC and can inhibit GC cell proliferation (52).

The estrogen receptor (ER) is a member of the nuclear receptor superfamily of ligand-activated transcription factors and plays an important role in BC (82). miR-873-5p inhibits ER activity by targeting CDK3, thereby inhibiting the growth of BC cells (11).

As a chemokine, the binding of CXCL16 to its sole receptor CXCR6 can involve biological activities such as cell adhesion (26) and anti-tumor immunity (27). Silencing CXCL16 can inhibit the proliferation and invasion of cancer cells by regulating the NF-κβ signaling pathway (83). Overexpression of miR-873-5p targets CXCL16 and suppresses the NF-κβ signaling pathway in PTC cells, thereby inhibiting PTC cell proliferation (44).

HDGF is a secreted growth factor (84), which can interact with the β-catenin pathway and promote cancer cell proliferation (85). Therefore, miR-873-5p targeted down-regulation of HDGF may inhibit EC cell proliferation through the β-catenin signaling pathway (36). DEC2 plays an important role in circadian rhythm, cell proliferation, and apoptosis, and is also closely related to tumor progression (14). In ESCA, miR-873-5p can inhibit ESCA cell proliferation by targeting the DEC2 gene, thereby affecting the circadian rhythm (14, 50).

### MiR-873-5p and Cell Migration, Invasion, and EMT

**Figure 5:**
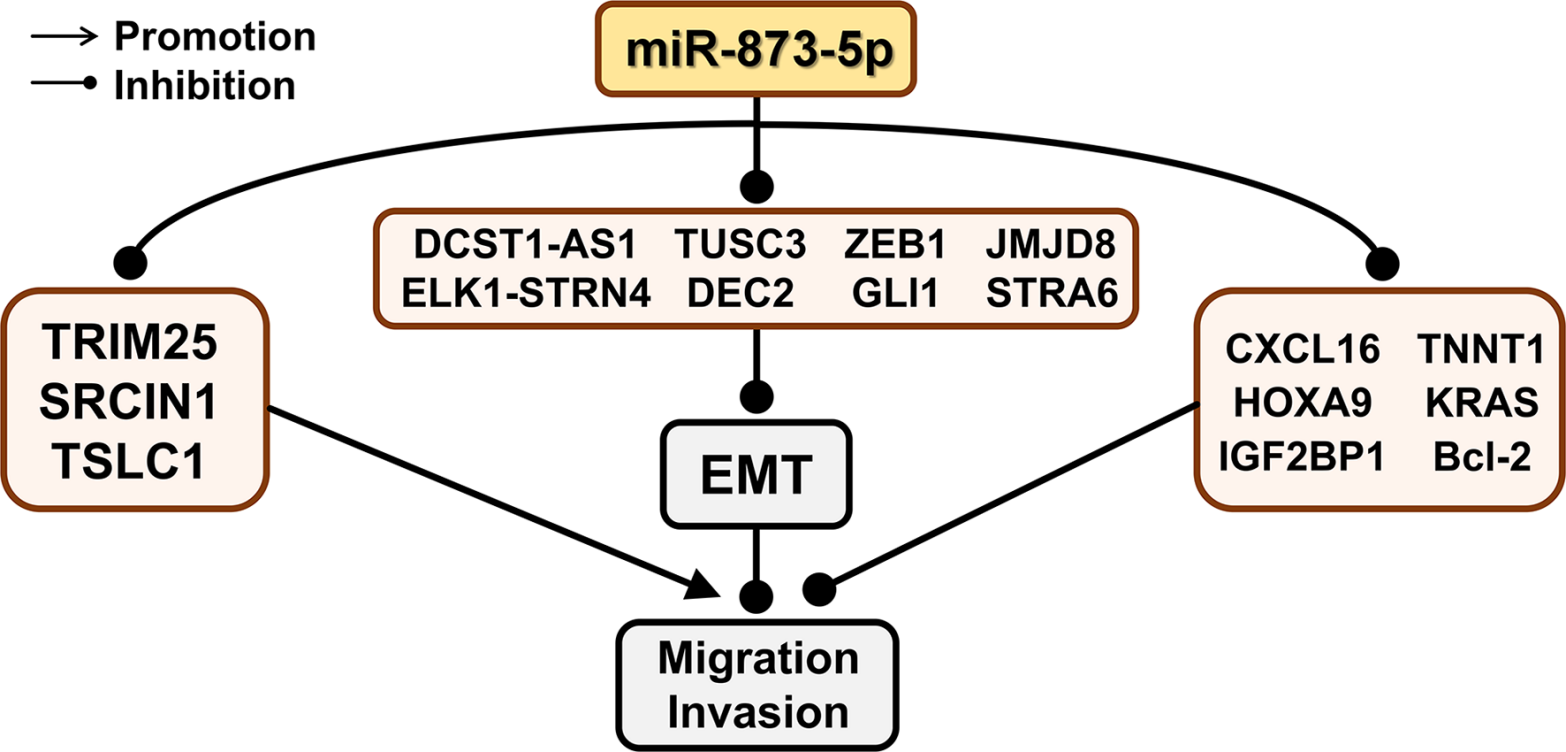
The effect of miR-873-5p target genes on EMT, migration, and invasion of cancer cells. miR-873-5p inhibits the EMT process by inhibiting the expression of ZEB1, TUSC3, DCST1-AS1, JMJD8, ELK1-STRN4, GLI1, STRA6, and DEC2. MiR-873-5p targets TRIM25, SRCIN1, and TSLC1, and promotes cell migration and invasion. In addition, miR-873-5p inhibits the migration and invasion of cancer cells by targeting CXCL16, TNNT1, IGF2BP1, Bcl-2, TNNT1, and HOXA9.

The migration and invasion of cancer cells are important for the progression of cancer. Epithelial cell-mesenchymal transition (EMT) is a process of epithelial cell changes, which is characterized by weak cell adhesion and enhanced migration ability (86). EMT is an important marker of cancer progression and metastasis of malignant tumors (87) (**Figure 5**).

In CRC HCT8 cells, the down-regulation of miR-873-5p corresponds to the up-regulation of ELK1 and STRN4, which leads to the down-regulation of E-cadherin and α-E-catenin and enhances EMT, and ultimately promotes the migration of CRC cells (47). ZEB1 is closely related to migration and EMT (88). In CRC, the up-regulation of miR-873-5p also corresponds to the down-regulation of ZEB1 expression, thereby significantly increasing the levels of E-cadherin, β-catenin, and ZO-1. This leads to a decrease in the levels of N-cadherin and vimentin, which changes the cell phenotype from EMT to MET, thereby inhibiting the EMT process of CRC cells (46). When miR-873-5p targets JMJD8, the expression of E-cadherin and cytokeratin is significantly increased, thereby weakening the EMT effect and inhibiting the migration and invasion of CRC cells (6). TUSC3 may change the EMT of CRC by regulating PI3K/Akt and WNT/β-catenin signaling pathways, thereby changing its metastasis and invasiveness (89). miR-873-5p can negatively regulate the expression of TUSC3, thereby inhibiting the EMT ability of CRC cells (49). TNNT1 is negatively correlated with the expression of E-cadherin in colon adenocarcinoma (90). miR-873-5p can regulate E-cadherin expression by targeting TNNT1, thereby inhibiting CRC cell migration and invasion (48).

When miR-873-5p targets to inhibit GLI1, the expression level of E-cadherin is significantly increased, while the levels of N-cadherin and vimentin are significantly reduced, thereby inhibiting the EMT process of CC cells (35). miR-873-5p can negatively regulate ULBP2 and activate immune cells, thereby reducing the invasion and metastasis of CC cells (34).

In GC cells, miR-873-5p can lead to the downregulation of N-cadherin and vimentin by inhibiting STRA6, thereby inhibiting the EMT process of GC cells, and cell metastasis and invasion (52).

LEF1 is an important transcription factor involved in the activation of the Wnt signaling pathway, which can promote the synthesis of mesenchymal fibronectin and EMT (91). When miR-873-5p binds to DCST1-AS1, the expression of LEF1 is up-regulated, and the EMT of TNBC cells is enhanced to promote cancer cell migration and invasion (38).

In ESCA, miR-873-5p can down-regulate the expression of DEC2, thereby inhibiting the effect of EMT and reducing the migration and invasion of ESCA cells (50, 92).

In PDAC and TNBC, miR-873-5p can target KRAS, thereby inhibiting cell migration and invasion through the ERK/AKT signaling pathway (39). The Wnt/β-catenin signaling pathway is a key mechanism for cell maintenance and development, including cell differentiation, migration, and invasion (93).

miR-873-5p can target HOXA9 and inhibit the migration and invasion of OS cells through suppressing the Wnt/β-catenin signaling pathway (43).

MTA-1 can promote cell metastasis through histone deacetylation and nucleosome remodeling (66). After miR-873-5p inhibits the expression of TRIM25, the function of MTA-1 is enhanced to promote the metastasis and invasion of HCC cells (8). TSLC1 is a specific tumor suppressor involved in cell adhesion and invasion (94). Therefore, in HCC, miR-873-5p can target TSLC1 to increase HCC cell adhesion, thereby promoting HCC cell migration (30).

SRCIN1 is the main regulator of E-cadherin (95), which can regulate the growth and movement of cell (96). miR-873-5p down-regulates the expression of SRCIN1, which can reduce cell adhesion and promote the migration of LUAD cell A549 (28).

IGF2BP1 can enhance the directionality of cell migration in a PTEN-dependent manner. miR-873-5p can down-regulate the expression of PTEN by targeting IGF2BP1, thereby inhibiting the migration ability of GM cells (42). Matrix metalloproteinases (MMP) have been shown to activate and regulate GM cell migration (97). Bcl-2 is an oncogene and it can promote the migration and invasiveness of GM cells by enhancing the activity of MMP (98). miR-873-5p can target Bcl-2 to enhance the activity of MMP and inhibit the migration and invasion of GM cells (9). MMPs are related to the development of cancer, which can promote the degradation of extracellular matrix and cell invasion and metastasis (99, 100). Overexpression of miR-873-5p can inhibit the expression of MMP1, MMP9, and MMP13 by down-regulating CXCL16, thereby inhibiting the migration and invasion of PTC cells (44).

### MiR-873-5p and Cell Stemness

Although cancer stem cells (CSCs) only account for a small part of cancer cells, they have the ability to self-renew (101). At present, CSC is considered to be the main factor leading to tumor recurrence and drug resistance (102).

Programmed cell death ligand 1 (PD-L1) is an immune checkpoint molecule and a ligand for PD-1 (103). The expression of PD-L1 is highly correlated with stemness-related genes in BC tissues and is overexpressed in basal BC. Therefore, PD-L1 may promote the stemness of BC cells (104, 105). PD-L1 can activate the PI3K/AKT and ERK signaling pathways in BC (106). And miR-873-5p can target PD-L1 and down-regulate its expression, and then inhibit the stemness of BC cells through the PI3K/Akt and ERK1/2 signaling pathways (10).

HuR is an RNA binding protein that can promote the progression of various tumors (107). HuR can directly bind and up-regulate CDK3 to promote the stemness of LCA (33). miR-873-5p can competitively bind to CDK3 with HuR and reduce CDK3 expression, thereby reducing the stemness of LCA cells (33).

Studies have found that ZIC2 may affect the occurrence and development of tumors through the AKT signaling pathway (108). Up-regulation of miR-873-5p can inhibit the expression of ZIC2 and disrupt the AKT signaling pathway, thereby inhibiting the stemness and tumorigenicity of NPC cells (109). Overexpression of miR-873-5p can silence PLEK2 and inhibit the self-renewal of PC stem cells through the PIK3/AKT signaling pathway, thereby inhibiting the development of PC (54).

### MiR-873-5p and Glycolysis

Tumor cells can change their metabolism to adapt to the challenging hypoxic environment (110). Intermediates in glycolysis can be used to meet the biosynthetic needs of rapidly growing tumors (111). AKT/rapamycin (mTOR) activation enables the continued growth and survival of tumor cells that rely on aerobic glycolysis, while the expression of NDFIP1 reduces the AKT/mTOR signaling pathway in cancer cells (5). In HCC, miR-873-5p inhibits the Warburg effect through the NDFIP1/AKT/mTOR axis, thereby inhibiting the aerobic glycolysis of HCC cells (5).

## The Role of miR-873-5p in Cancer Treatment

**Figure 6:**
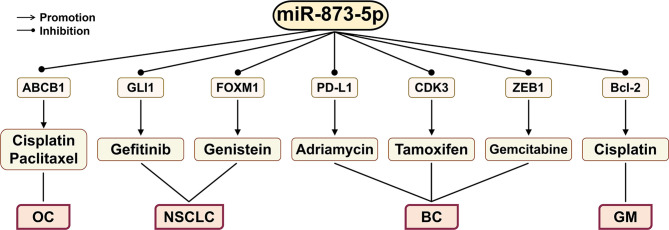
The effect of miR-873-5p on the efficacy of different cancer chemotherapy drugs through its target genes. miR-873-5p inhibits the expression of ABCB1, Bcl-2, GLI1, FOXM1, PD-L1, CDK3, and ZEB1, thereby improving the inhibitory effects of various anticancer drugs on cancer cells. OC, ovarian cancer; GM, glioblastomas; LC, lung cancer; BC, breast cancer.

Gefitinib (EGFR-TKI) can reduce viability and proliferation of cancer cells and angiogenesis in NSCLC (**Figure 6**). However, the resistance of cancer cells to gefitinib has greatly limited its clinical application (4, 112, 113). The enhancement of the GLI1 expression can increase the radiation resistance of NSCLC cells. When GLI1 is silenced, gefitinib can significantly reduce the growth of NSCLC cells (114, 115). The down-regulation of GLI1 by miR-873-5p can reduce the resistance of NSCLC cells to gefitinib, thereby causing NSCLC PC9 cell apoptosis (4).

The main treatments for BC include surgery, targeted therapy, radiotherapy, and chemotherapy. For TNBC, chemotherapy is the only treatment (10). CSCs may contribute to the chemoresistance of cancer (116). By activating the PI3K/Akt and ERK1/2 signaling pathways, the PD-1/PD-L1 axis can promote the stemness and drug resistance of BC cells. miR-873-5p targeted inhibition of PD-L1 expression can attenuate the resistance of BC cells to Adriamycin (10). In addition, miR-873-5p may also inhibit ERa phosphorylation by targeting CDK3, thereby restoring the sensitivity of BC drug-resistant cells to tamoxifen (11).

Norbiliin (NCTD) is a dimethyl analog of phthalazine, which can inhibit the biological functions of cell proliferation and angiogenesis in a variety of cancers (117–119). NCTD can overcome tamoxifen resistance by targeting the miR-873-5p/CDK3 axis in BC cells (120).

Gemcitabine is a chemotherapy drug that is derived from deoxycytidine and is commonly used to treat BC patients (121). ZEB1 plays a key role in promoting the development of CSCs, and its overexpression is related to cancer chemoresistance (15). miR-873-5p can bind to the 3’-UTR of ZEB1 to directly inhibit its expression, thereby enhancing the cell growth inhibition induced by gemcitabine treatment (37).

Ovarian cancer (OC) is mostly treated with cisplatin and paclitaxel, but OC cancer cells often develop resistance to these drugs (122). The ABC superfamily transporter and P-glycoprotein (MDR1) play a key role in the multidrug resistance (MDR) of cancer. They can mediate the outflow of various chemical drugs, such as anticancer drugs (123–125). Overexpression of miR-873-5p increases the sensitivity of OC cells to cisplatin and paclitaxel by targeting ABCB1 to down-regulate the expression of MDR1 (126).

GM is the most common primary brain tumor in adults, and cisplatin is currently a chemical drug widely used to treat GM (127, 128). A study has found that inhibiting the expression of Bcl-2 can enhance the sensitivity of GM to cisplatin (129). miR-873-5p can enhance the sensitivity of GM cells to cisplatin by targeting Bcl-2 (9).

In addition, genistein is a soy-derived isoflavone that can play a beneficial role in cancer treatment (130). Genistein can inhibit the progression of NSCLC by regulating the circ_0031250/miR-873-5p/FOXM1 axis (131).

## The Regulation of miR-873-5p in Human Cancer

**Figure 7:**
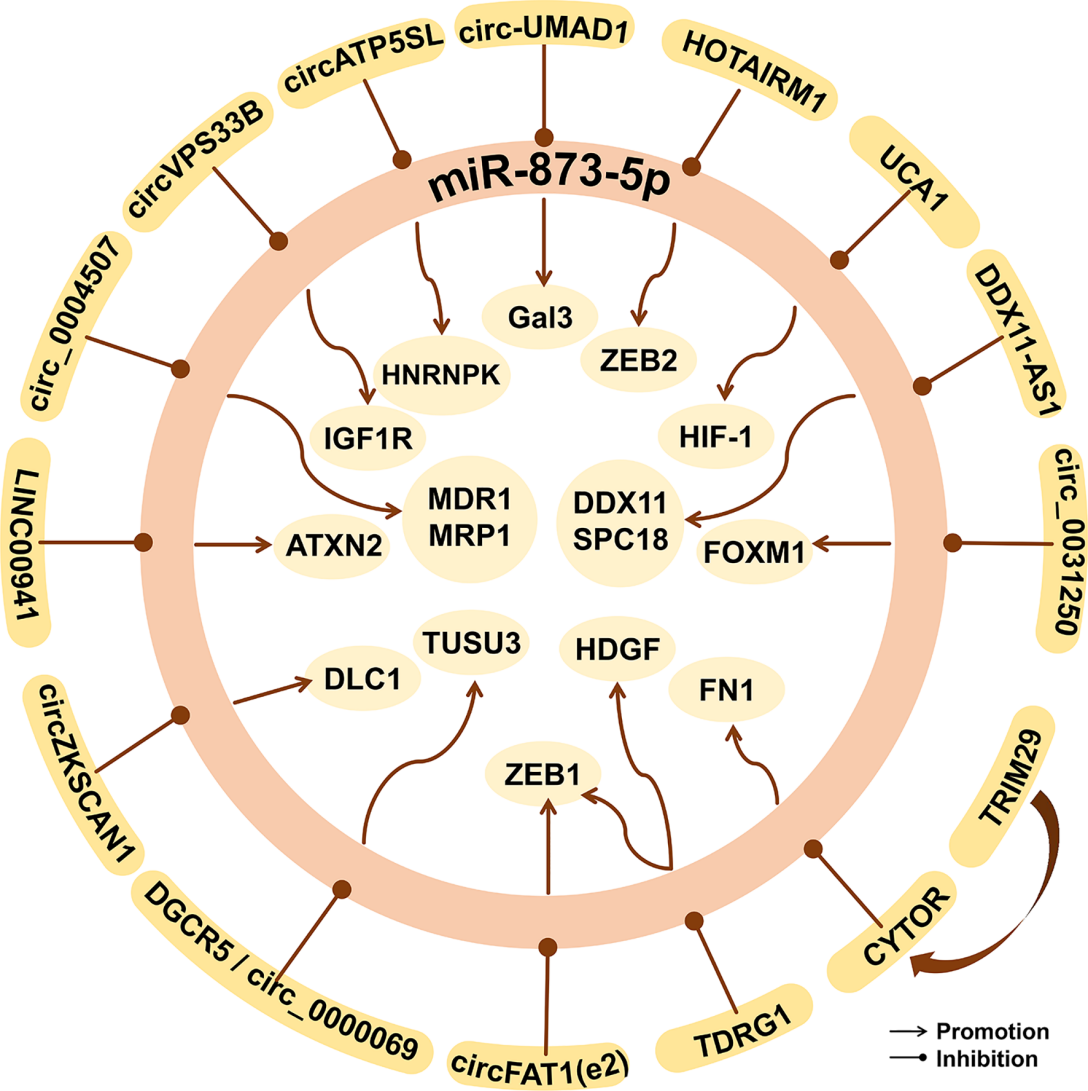
The epigenetic factors of miR-873-5p in human cancer. miR-873-5p can be targeted and regulated by lncRNAs, circRNAs, and other proteins, thus affecting downstream gene expression and playing an important role in cancer.

Current studies have found that methyltransferase, circRNA, and lncRNA are involved in the regulation of miR-873-5p in human cancer (**Figure 7**).

CircRNA is a new type of non-coding RNA that can bind miRNAs to stop their regulation of target genes (132). Hsa_circ_0000069 can sponge miR-873-5p, which can promote the expression of TUSC3, thereby promoting the proliferation, migration, and invasion of CC cells (133). In Neuroblastoma, circDGKB can sponge miR-873-5p to increase the expression of ZEB1 and GLI1, and promote the occurrence and development of cancer (134). circ-UMAD1 can sponge miR-873-5p, thereby up-regulating the expression of Galectin-3 and inducing lymphatic metastasis of PTC (135). circFAT1(e2) can promote the proliferation, metastasis, and invasion of PTC cells by inhibiting the miR-873-5p/ZEB1 axis, thereby exerting a carcinogenic effect (136). Knockout of circ_0004507 can up-regulate the expression of miR-873-5p and inhibit the progression of laryngeal cancer (137). circ_0031250 can promote the proliferation, migration, and invasion of NSCLC cells by inhibiting the miR-873-5p/FOXM1 axis (131). circZKSCAN1 can inhibit the progression, proliferation, migration, and invasion of HCC by down-regulating the miR-873-5p/DLC1 axis, thereby hindering the occurrence and development of HCC (138). Infant hemangioma (IH) is one of the most benign endothelial tumors in infants and young children. circATP5SL can eliminate the inhibition of IGF1R by sponging miR-873-5p, thereby promoting IH cell invasion, proliferation, and migration (139). circVPS33B accelerates tumor cells’ proliferation, migration, and growth by down-regulating the miR-873-5p/HNRNPK axis in invasive GC (140).

LncRNA MCF2L-AS1 can promote CSC-like characteristics of NSCLC cells by down-regulating the expression level of miR-873-5p, thereby exerting carcinogenic effects (141). YY1 is a member of the YY family. It is a zinc finger protein and is overexpressed in a variety of cancers (142). YY1 can down-regulate the level of miR-873-5p, thereby activating the PI3K/AKT and ERK signaling pathways, thereby promoting the stemness of cancer cells (58). LncRNA CYTOR can regulate the expression of genes in the nucleus, thereby participating in the occurrence and development of cancers such as CRC (143). By up-regulating lncRNA CYTOR, TRIM29 inhibits pre-mir-873-5p to produce miR-873-5p, thereby up-regulating FN1 and promoting the migration and invasion of PTC cells (144).

The expression of lncRNA DGCR5 is significantly reduced in LC. DGCR5 shares the same binding site of miR-873-5p with TUSC3 (145). Ki-67 and MMP-3, MMP-9 are the markers of cell proliferation, cell migration, and invasion (100, 146). The binding of DGCR5 to miR-873-5p reduces the expression of TUSC3, Ki-67, MMP-3, and MMP-9, and thus decreases the proliferation and migration ability of LC cells (145). LncRNA TDRG1 is a proto-oncogene for CC (147) and endometrial cancer (148). The expression of lncRNA TDRG1 is up-regulated in human GC tissues and is related to the clinical prognosis of GC patients (149). As an important regulator of cancer, HDGF can be down-regulated through the EMT signaling pathway and the MMP-2 and MMP-9 signaling pathways (150). TDRG1 can target the miR-873-5p/HDGF axis, thereby promoting the tumor phenotype of GC cells (149). In addition, TDRG1 up-regulates the expression of ZEB1 by targeting miR-873-5p, thereby promoting tumorigenesis and the development of NSCLC cell lines (151). LncRNA HOTAIRM1 inhibits the miR-873-5p expression and promotes the expression of ZEB2 in GM, thereby inhibiting tumor cell apoptosis (74).

Competitive endogenous RNA (ceRNA) is considered to be a mechanism in post-transcriptional regulation and is related to tumor progression (152, 153). In OS, miR-873-5p targets to inhibit the expression of DDX11, and thus reduces the expression of MMP2, MMP9, N-cadherin, but increases the expression of E-cadherin, thereby inhibiting the migration and EMT process of OS cell lines (154). LncRNA DDX11-AS1 is up-regulated in GC tissues and cell lines, and its expression increases with the development of TNM stages and lymph node metastasis (155). LncRNA DDX11-AS1 as a ceRNA can bind to miR-873-5p and up-regulate the expression of DDX11 in OS and SPC18 in GC, thereby promoting the occurrence and development of OS (154) and GC (155).

Single nucleotide polymorphisms (SNPs) can change the secondary structure of lncRNA, thereby affecting the interaction between lncRNA and its interacting miRNA, and ultimately increasing the risk of cancer (156). The rs12982687 site of lncRNA UCA1 can affect the binding of miR-873-5p, thereby increasing the function of HIF-1 signal transduction, promoting the proliferation and migration of CRC cells (157).

## MiR-873-5p and the Prognosis of Cancer Patients

At present, many studies have found that miR-873-5p is significantly related to the prognosis of cancer patients (**Table 2**). Compared with normal tissues, the expression level of miR-873-5p is increased not only in HCC tissues but also in advanced HCC. Increased expression of miR-873-5p in HCC is positively correlated with lymph node metastasis and metastasis stage, but negatively correlated with tumor differentiation, indicating that miR-873-5p may be related to the aggressiveness and poor prognosis of HCC (5). In addition, low expression of miR-873-5p is associated with poor prognosis of LUAD (29).

**Table 2:** The prognostic value of miR-873-5p in different cancers.

Cancer	Materials	Results	Reference
HCC	86 HCC tissues and 86 matched non-tumor tissues	The level of miR-873-5p in advanced liver cancer is higher than that in peripheral liver cancer. The overall survival and recurrence time of HCC patients with low miR-873-5p expression levels are much longer than those of HCC patients with high miR-873-5p expression, which indicates that higher miR-873-5p expression is related to the poor prognosis of HCC.	(5)
CRC	50 CRC tissues and 50 adjacent normal tissues; 96 CRC tissues and 96 adjacent normal tissues	The level of miR-873-5p is negatively correlated with the degree of malignancy of CRC. Patients with high miR-873-5p levels have a longer overall survival rate than patients with low miR-873-5p levels, which indicates that lower miR-873-5p expression is related to a poor prognosis of CRC.	(6, 49)
LUAD	481 LUAD tissues and 47 normal tissues	miR-873-5p is an independent prognostic factor of LUAD. The high expression of miR-873-5p indicates that the survival rate of LUAD patients is lower.	(29)
GC	80 GC tissues and 80 adjacent normal tissues	Low miR-873-5p is associated with tumor enlargement in GC patients, advanced T-grade, and poor histological type, and predicts poor OS and DFS.	(52)

LUAD, lung adenocarcinoma; HCC, hepatocellular carcinoma; CRC, carcinoma of colon and rectum; GC, gastric cancer; DFS, disease-free survival; OS, overall survival.

Decreased expression of miR-873-5p is an indicator of poor prognosis in CRC patients (49). In addition, the level of miR-873-5p is negatively correlated with the degree of malignancy of CRC, and high levels of miR-873-5p are significantly correlated with a longer overall survival rate of patients (6). In GC, low expression of miR-873-5p is associated with large tumors, advanced T grade, poor histological type, poor overall survival, and short recurrence-free survival (52). In CC, the overall survival rate of patients with low miR-873-5p expression is lower than that of patients with high miR-873-5p expression (34).

## The Role of miR-873-3p in Cancer

**Figure 8:**
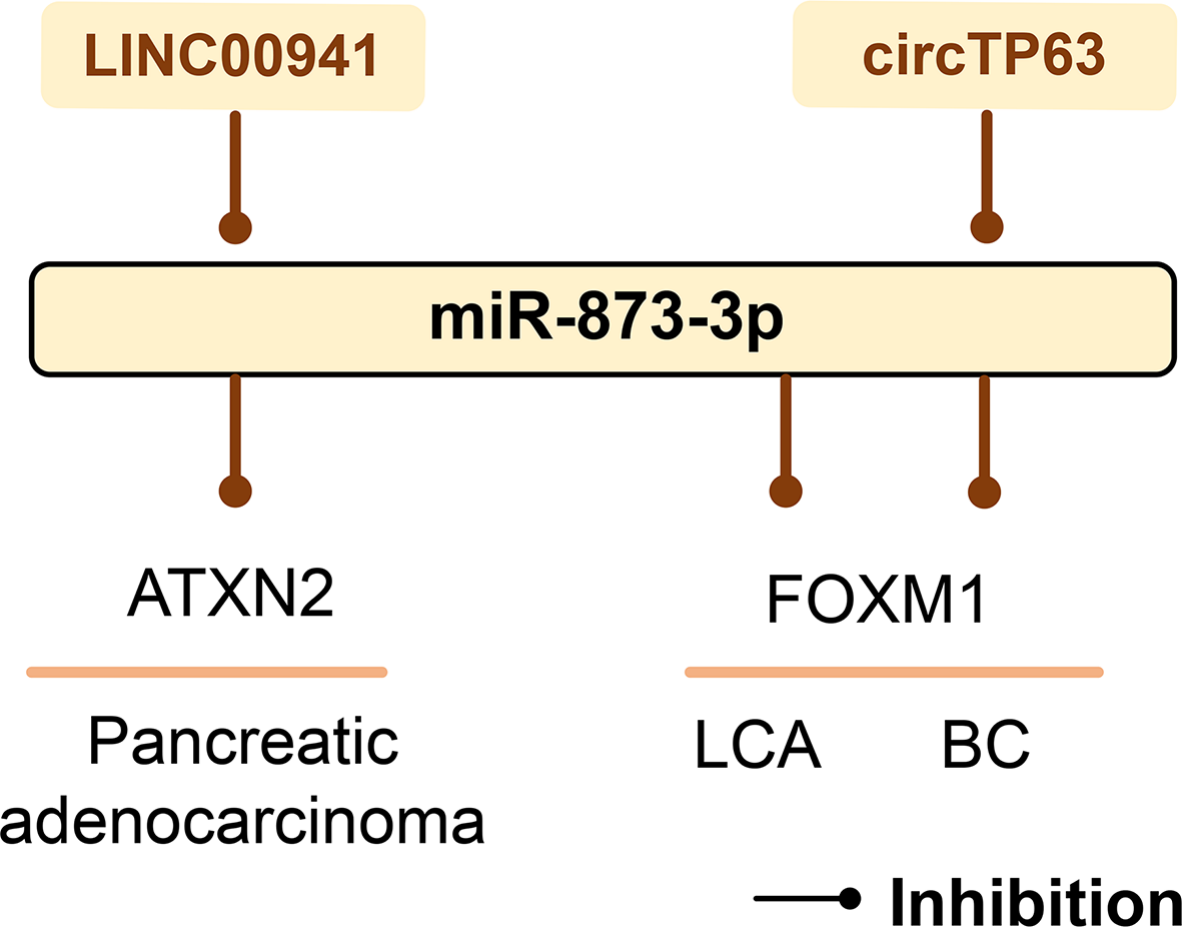
The role of miR-873-3p in human cancer. mir-873-3p plays an important role in pancreatic adenocarcinoma, LCA, and BC by regulating target genes. LINC00941 and circTP63 can sponge miR-873-3p and thus affect cancer development. LCA, lung cancer; BC, breast cancer.

Proliferation-specific fork head box m1 (FOXM1) has been identified as an important cell cycle regulator, which can control the transition of cells from G1 to S phase and cell progression to M phase (158). miR-873-3p can significantly reduce the mRNA and protein levels of FOXM1. Therefore, miR-873-3p targets FOXM1 to inhibit LCA cell proliferation through its cell cycle regulation function (159). In BC, circTP63 binds to miR-873-3p and prevents its targeted inhibition of FOXM1, thereby inducing the progression and growth of estrogen receptor-positive BC (160). LINC00941 up-regulates the expression of ATXN2 by competitively binding miR-873-3p, stimulates the proliferation and metastasis of pancreatic adenocarcinoma, and promotes its occurrence and development (161) (**Figure 8**).

## Conclusions and Perspectives

miR-873-5p is widely involved in the progression of cancer, its expression is dysregulated in most cancer tissues and cell lines. Besides, the target gene of miR-873-5p has a series of molecular regulation functions, such as catalytic activity, transcription regulation, and binding. In cancer, miR-873-5p affects cancer development through the PIK3/AKT/mTOR, Wnt/β-Catenin, NF-κβ, MEK/ERK signaling pathways. miR-873-5p involves a variety of biological processes through the regulation of target genes, such as cell proliferation and apoptosis, EMT, cell migration and invasion, cell cycle, and cell stemness. miR-873-5p can also inhibit or promote the effects of cancer drugs by regulating its target genes. miR-873-5p can also be used as a specific diagnostic and prognostic indicator for various cancers. Finally, this review also summarizes epigenetic regulatory factors of miR-873-5p, including lncRNA, circRNA, methyltransferase, etc., which are also involved in the occurrence and development of various cancers.

However, there are still many deficiencies in the research on miR-873-5p. First of all, current studies have shown that miR-873-5p is dysregulated in 18 kinds of cancers, and it can cause cancer or suppress cancer. However, existing studies have not proven that miR-873-5p is cancer-specific. This will limit the application of miR-873-5p for cancer diagnosis, and it needs to be further explored. Second, the specific mechanism of miR-873-5p in some cancers has not been studied. Besides, more preclinical studies and clinical trials are needed to explore the effects of miR-873-5p on the efficacy of anticancer drugs. Finally, most studies are involved with miR-873-5p, and the research on miR-873-3p is very lacking.

Here we show that miR-873-5p plays a significant role in the initiation and progression of key biological and pathological processes in human cancers. Therefore, miR-873-5p can be the main research focus in the fight against human cancers. This review mainly summarizes the research progress of miR-873-5p in human cancers, which will expand our understanding of the molecular and cellular biological mechanisms of miR-873-5p.

## Author Contributions

SD, ZH, and YZ conceived the review. CZ and YZ collated and analyzed the literature. SD and YZ helped complete diagrams and writing papers. All authors contributed to the article and approved the submitted version.

## Conflict of Interest

The authors declare that the research was conducted in the absence of any commercial or financial relationships that could be construed as a potential conflict of interest.

## Publisher’s Note

All claims expressed in this article are solely those of the authors and do not necessarily represent those of their affiliated organizations, or those of the publisher, the editors and the reviewers. Any product that may be evaluated in this article, or claim that may be made by its manufacturer, is not guaranteed or endorsed by the publisher.
